# Effects of chitosan on plant growth under stress conditions: similarities with plant growth promoting bacteria

**DOI:** 10.3389/fpls.2024.1423949

**Published:** 2024-11-08

**Authors:** Maura Rojas-Pirela, Petronia Carillo, Cristóbal Lárez-Velásquez, Gianfranco Romanazzi

**Affiliations:** ^1^ Laboratorio de Enzimología de Parásitos, Departamento de Biología, Facultad de Ciencias, Universidad de Los Andes, Mérida, Venezuela; ^2^ Dipartimento di Scienze e Tecnologie Ambientali Biologiche e Farmaceutiche, Università degli Studi della Campania, Caserta, Italy; ^3^ Grupo de Polímeros, Departamento de Química, Universidad de Los Andes, Mérida, Venezuela; ^4^ Department of Agricultural, Food and Environmental Sciences, Marche Polytechnic University, Ancona, Italy

**Keywords:** biostimulants, chitosan, induced resistance, plant growth-promoting bacteria, stress 1

## Abstract

The agricultural use of synthetic pesticides, fertilizers, and growth regulators may represent a serious public health and environmental problem worldwide. All this has prompted the exploration of alternative chemical compounds, leading to exploring the potential of chitosan and PGPB in agricultural systems as a potential biotechnological solution to establish novel agricultural production practices that not only result in fewer adverse impacts on health and the environment but also improve the resilience and growth of the plants. In this work, an analysis of the impact of plant growth-promoting bacteria (PGPB) and chitosan on plant growth and protection has been conducted, emphasizing the crucial bioactivities of the resistance of the plants to both biotic and abiotic stressors. These include inducing phytohormone production, mobilization of insoluble soil nutrients, biological nitrogen fixation, ethylene level regulation, controlling soil phytopathogens, etc. Moreover, some relevant aspects of chitin and chitosan are discussed, including their chemical structures, sources, and how their physical properties are related to beneficial effects on agricultural applications and mechanisms of action. The effects of PGPB and chitosan on photosynthesis, germination, root development, and protection against plant diseases have been compared, emphasizing the intriguing similarities and synergistic effects observed in some of these aspects. Although currently there are limited studies focused on the combined application of PGPB and chitosan, it would be important to consider the similarities highlighted in this work, and those that may emerge in future studies or through well-designed investigations, because these could permit advancing towards a greater knowledge of these systems and to obtain better formulations by combining these bioproducts, especially for use in the new contexts of sustainable agriculture. Thus, it seems feasible to augur a promising near future for these combinations, considering the wide range of possibilities offered by chitinous biomaterials for the development of innovative formulations, as well as allowing different application methods. Likewise, the studies related to the PGPB effects on plant growth appear to be expanding due to ongoing research to test on plants the impacts of microorganisms derived from different environments, whether known or recently discovered, making it a very exciting field of research.

## 1 Introduction

Bacteria present in the soil can have beneficial, neutral, or harmful effects on plants. Many rhizosphere bacteria have additionally demonstrated distinctive abilities to promote plant growth in environments that generate strong abiotic stresses, such as arid or seasonally dry regions, whose soils usually experience drought, salinity, or high temperatures (Khan et al., 2020). Likewise, some bacteria can promote plant growth in other extreme conditions, i.e., regions of high altitudes and low temperatures (Kadıoglu et al., 2018). Plant-associated bacteria that have a positive influence on the growth and development of plants are collectively called plant growth-promoting bacteria (Khatoon et al., 2022; Ajijah et al., 2023). They can promote plant growth directly, playing an active role in nutrient solubilization (e.g., P, K, Fe, Ca, and Mg) or atmospheric nitrogen fixation thus facilitating their acquisition, or producing phytohormones able to stimulate plant growth and development. In addition, they can act as biocontrol agents antagonizing other microorganisms that affect plant health and vigor. Among the responses they can generate are:

Mobilization of insoluble nutrients towards the rhizosphere through the secretion of compounds such as the so-called siderophores, which act as chelating agents for metals such as Fe (e.g.), making them more available for plants (He et al., 2020). They are produced especially by Gram-negative bacteria and can be classified according to their iron-binding moieties: carboxylate, catecholate, hydroxamate, and phenolate moieties). Siderophores which are specific for Fe(III), by forming stable aqueous complexes with it, display a similar or higher affinity for Mn(III) and other transition metals like Zn^2+^ or Cu^2+^ (Li et al., 2020b). In contaminated soils, siderophores can also chelate Cd^2+^ or other toxic metals like Al^3+^, Co^2+^, Pb^2+^, etc., reducing their bioavailability for plants and favoring their growth in these less favorable conditions (Guo et al., 2020). Strikingly, a similar mode of action has also been observed for materials with chelating activity such as chitosan (Heidari et al., 2020), with metal chelation standing out as a proposed mechanism to explain the protective role of chitosan favoring plant growth (Chakraborty et al., 2020). Chitosan metal-binding capacity increases at higher pH levels due to unprotonated amine groups, facilitating interaction with and absorption of metal ions (Abdellatef et al., 2022). On the other hand, the effects of chitosan as a chelating agent on plant growth under stress conditions, such as in the case of plants growing in soil contaminated by metals, are not easy to predict and depend on many factors. A good example of this situation is provided by the results obtained during the application of foliar treatments with chitosan to sunflower plants growing in soils “contaminated” separately with lead and cadmium (added as cations in aqueous solution and in equivalent quantities). While one of the treatments attenuates the effects of both cations on root length to the same extent (73%), a similar treatment, using a higher dose of chitosan, produces a lower attenuation of the effect of Pb (31%) than for Pb (75%) (Cartaya-Rubio et al., 2023).

Nitrogen-fixing bacteria, forming symbiotic relationships with agricultural plants and able to reduce atmospheric nitrogen (N_2_) to ammonium in a reaction catalyzed by nitrogenase, belong to various genera, including *Azotobacter*, *Azospirillum*, *Bacillus*, *Bradyrhizobium*, *Pseudomonas*, and *Rhizobium* (Zaidi et al., 2015). In rhizobia, flavonoid compounds released by legume roots interact specifically with the transcriptional regulator NodD whose activation triggers the expression of nodulation genes necessary for synthesizing lipo-chitooligosaccharides known as Nod factors. These latter, chitin-derivatives obtained by replacing the N-acetyl groups with fatty acids at the reducing ends, act as plant-specific bioactive molecules (Lerouge et al., 1990) signaling the establishment of the symbiotic association with legumes (Long, 1996). It is also interesting that arbuscular mycorrhizal fungi use these same types of molecules to initiate their symbiotic relationship with plants (Limpens et al., 2015).

Induction of the production of phytohormones such as gibberellins (GAs) and auxins, among which indole-acetic acid (IAA) stands out. In some cases, such as with *Pseudomonas monteilii*, it has been found that induction of IAA synthesis can be increased when other materials are added, such as chitosan nanoparticles (Panichikkal et al., 2022). The excessive application of chitosan in the rhizosphere of *Arabidopsis* has been also observed to cause stress in these plants, which is made evident by the arrest in the root growth due to the accumulation of auxins, mainly IAA (Lopez-Moya et al., 2019). Therefore, it is extremely important to control the concentrations of chitosan used in agricultural practices to inhibit the growth of plant pathogens, while simultaneously favoring the proliferation of beneficial organisms without compromising plant growth.

Modulation of ethylene (C_2_H_4_), a gaseous phytohormone playing a pivotal role in regulating several aspects of plant development and physiology, such as seed germination, cell expansion, leaf and petal abscission, fruit ripening and abscission, organ senescence, and responses to environmental stresses (Iqbal et al., 2017). Moreover, ethylene influences tissue differentiation, root and shoot formation, and branching and root elongation (Glick, 2014). Thus, during the establishment of symbiotic plant-rhizobium or plant-mycorrhizal fungus relationships, a local decrease in ethylene concentration may occur facilitating symbiosis (Gamalero and Glick, 2015). On the other hand, chitosan has also been shown to have a regulatory effect on the production of ethylene in plants, in some stages of their agricultural production (Czékus et al., 2021; Prusky and Romanazzi, 2023). Thus, its application has a notable influence on the maturation of some climacteric fruits, both in pre- and post-harvest treatments. For instance, the ethylene concentration in banana (*Musa acuminata*) fruits sharply increases at the onset of their climacteric period, leading to a rapid ripening process with the consequent decrease in fruit shelf life. However, the postharvest application of chitosan as an edible coating on fruits allows them to extend their shelf life and maintain their sensory quality (Lustriane et al., 2018; Romanazzi and Moumni, 2022). Likewise, it has recently been found that a similar treatment with chitosan 1.25% w/v on banana fruits, with storage for 11 days, led to a distinctive accumulation of the metabolite 1-aminocyclopropane-1-carboxylic acid (ACC), the immediate precursor of ethylene, which is responsible for the ripening process of climacteric fruit (Parijadi et al., 2022).

Biological control of soil plant pathogens, competing with them for space and nutrients, either by sequestering essential metals for their development (Olanrewaju et al., 2017), or by releasing substances toxic to them, which may include enzymatic proteins such as chitinases (Velásquez et al., 2019; Liu et al., 2019a), or antimicrobial peptides such as bacteriocins, i.e*.*, turicin (Cesa-Luna et al., 2020), or other antibiotics (Matilla and Krell, 2017). On the other hand, one of the control mechanisms of phytopathogenic microorganisms usually associated with chitosan has been precisely the chelation of metals necessary for plant pathogens, especially those associated with molecules present in the cell walls of bacteria (Chandrasekaran et al., 2020).

Similarly, and as briefly outlined in the previous part, chitosan and some of its related materials can also produce stimulating effects on plant growth without compromising environmental sustainability (Shahrajabian et al., 2021), including in many cases a significant improvement in plant resilience under stress conditions (Hidangmayum et al., 2019; Malerba and Cerana, 2020).

Thus, in general terms, it has been stated that both chitosan and PGPB have adequate potential to enhance agricultural productivity in many current stressed environments and in future more extreme scenarios that are predicted due to climate change. In this work, a review of different studies related to the effects of PGPB and chitosan (usually applied independently) on plant growth is carried out. It is intended to organize information that can help to justify some similarities observed in the bioactivities of PGPB and chitosan, which had gone unnoticed. Thus, the knowledge derived from studies of this type can help develop strategies that use these combined systems, which should be very useful in the new contexts of sustainable agriculture, especially under stress conditions.

## 2 Chitosan and its beneficial effects on plants

**Figure 1:**
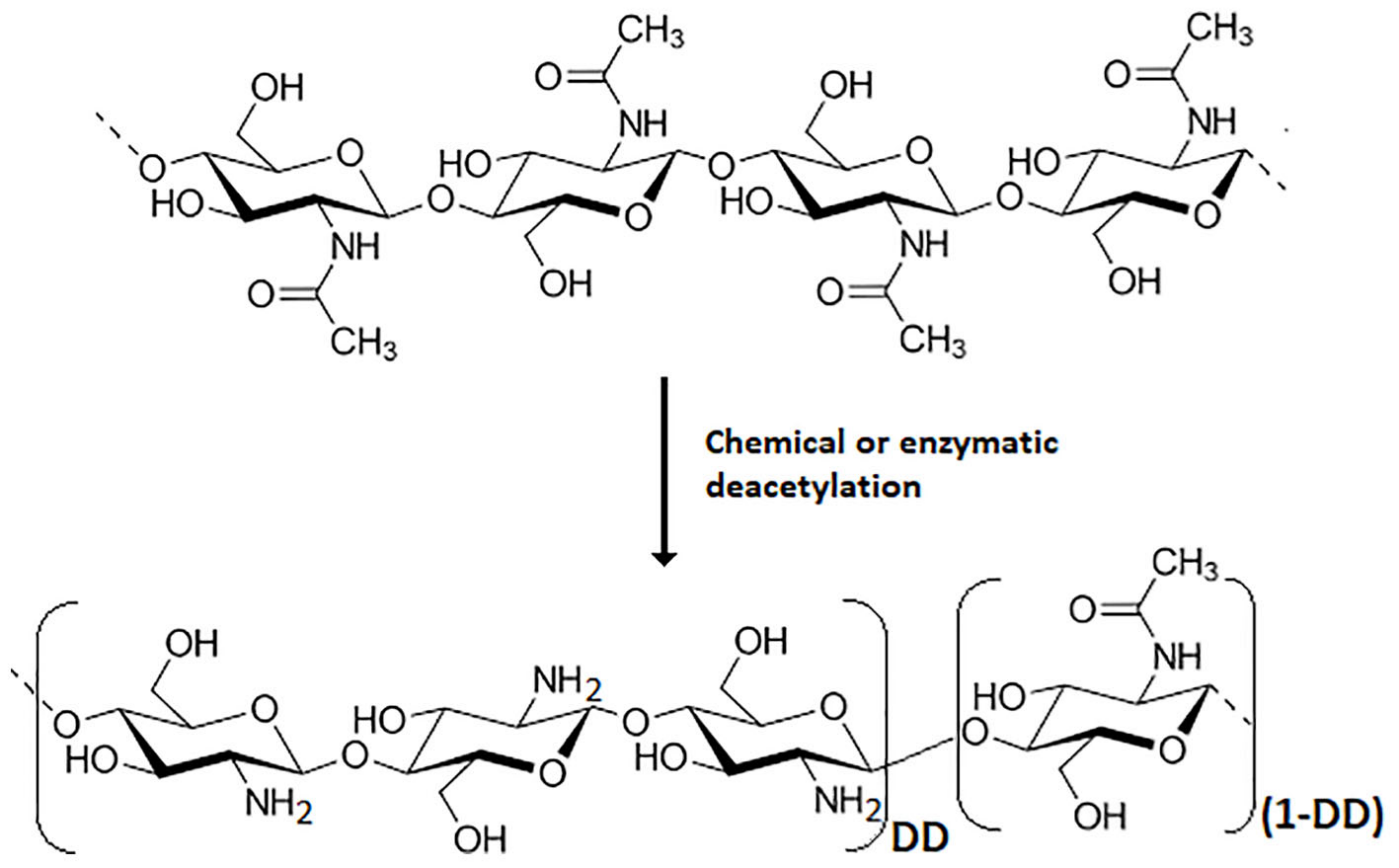
Chemical structure of chitin and its usual deacetylation reactions to obtain chitosan, which is defined by a deacetylation degree (DD) ≥ 50%.

**Figure 2:**
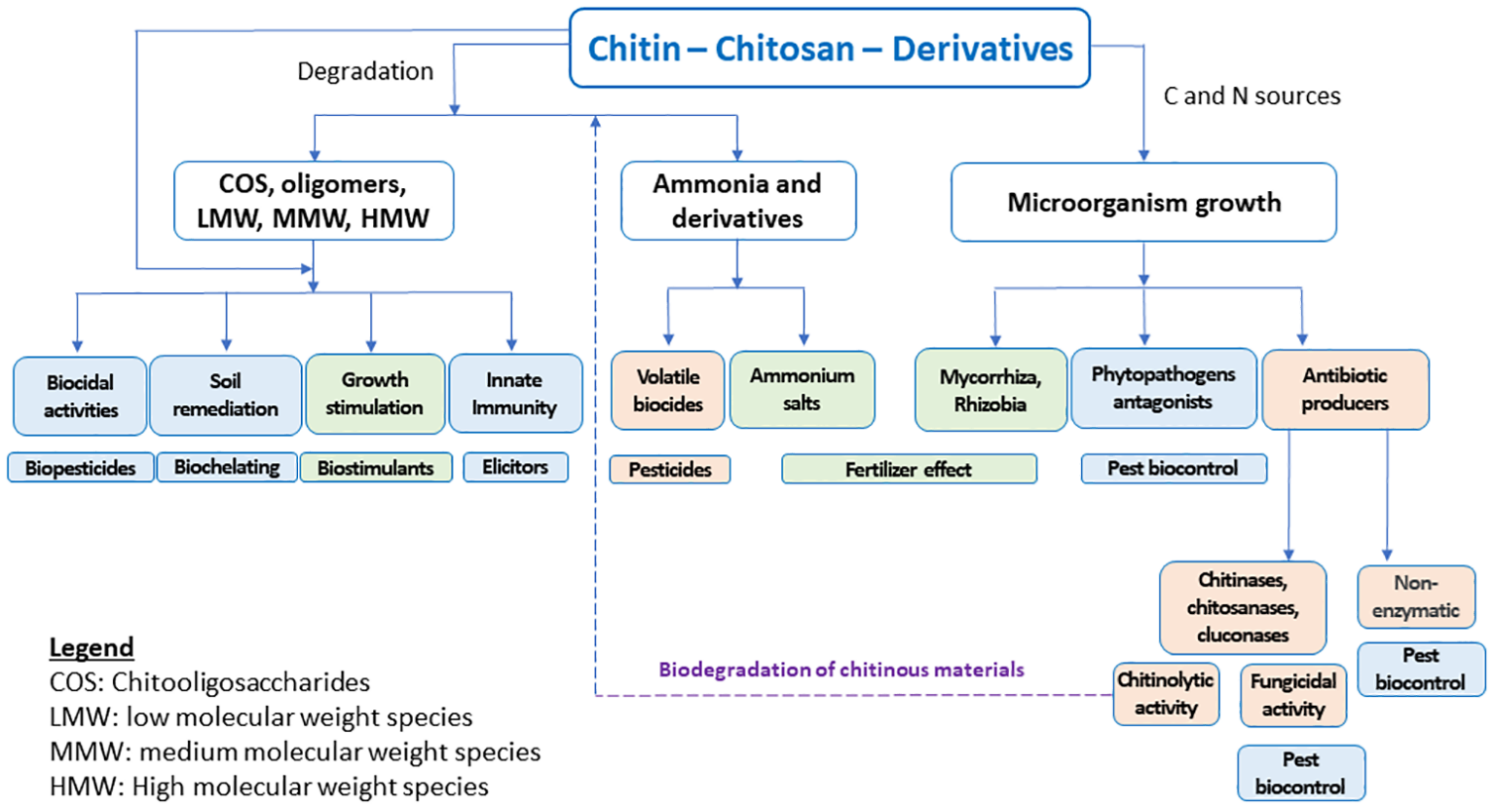
The main modes of action associated with the protection and plant growth provided by chitinous materials.

Chitosan is the main derivative of chitin, one of the more abundant natural biopolymers. Chitin is present in a wide diversity of organisms, including insects, crustaceans, mollusks, fishes, fungi, algae, etc (Lárez-Velásquez, 2023), and its deacetylation process yields chitosan (**Figure 1**). The beneficial effects of chitin and chitosan on agricultural uses have been known for a long time (Mitchell and Alexander, 1961; Nichols and Hadwiger, 1979). The main modes of action of these biopolymers in agricultural applications have been profusely reviewed (Velásquez et al., 2019; Riseh et al., 2022; Boamah et al., 2023) and some attempts are being made to organize the accumulated knowledge on the subject (Velásquez et al., 2019; Shamshina et al., 2020). **Figure 2** schematically shows the main proposed modes of action by which chitinous materials promote beneficial effects in plants.

In general, there are three types of factors that affect the bioactivity of chitosan: (a) those related to the chitosan molecules themselves such as the source and method employed to obtain it, the molecular weight (MW), the degree of deacetylation (DD), the acetylation pattern (AP), the polyelectrolyte associated counterion, the concentration, application method), (b) those associated with the kind and development stage of the pathogenic organism subjected to its action (bacteria, virus, fungi, insects) and (c) those that have to do with the environment where the processes are carried out (pH, temperature, ionic strength, presence of others chemical substances or live organisms, etc.). A few examples of proven agricultural applications, and the underlying mechanisms of action, are the following:

Phytopathogen control: Chitosan biocidal activity against phytopathogens has been amply proven and reviewed (Ke et al., 2021; Riseh et al., 2022), with different mechanisms having been proposed to explain its effects. Its applications have been studied to control diseases caused by viruses, bacteria, fungi, insects, etc., and its biocidal action has been found to depend largely on its cationic polyelectrolyte properties (acid medium). The main mechanisms proposed for its bioactivities concerning diseases caused by fungi are the disruption or alteration of the fungal plasma membrane, the alteration of gene expression, the inhibition of RNA and protein synthesis, and the blocking of Ca^2+^ channel (Lárez-Velásquez and Rojas-Avelizapa, 2020; Karamchandani et al., 2022). Chitosan control of bacterial phytopathogens has also been profusely proven (Badawy and Rabea, 2016; Khairy et al., 2022), with reports indicating higher effectivity for Gram-negative than for Gram-positive bacteria (Tarakanov et al., 2023).

Soil conditioning and bioremediation: Chitin is a recognized amendment that, in addition to providing nitrogen, can help in soil conditioning by freeing it from nematodes, either by promoting the growth of chitinolytic microorganisms that feed on their eggs or by killing them with the ammonia, or its derivatives, released during its decomposition (Sharp, 2013). In turn, metal chelation stands out as one of the proposed mechanisms to explain the protective role of chitosan on plant growth (Chakraborty et al., 2020), including phytoremediation species (Shirkhani et al., 2021).

Biostimulation and preservation of seeds: chitosan-based coatings have been applied to seed protection (Moumni et al., 2023). This type of application is not limited only to taking advantage of its fungicidal activity (Chookhongkha et al., 2013), or its bioactivity as a germination elicitor (Khaptsev et al., 2021), but can additionally serve as a vehicle for the controlled release of other agrochemicals (Godínez-Garrido et al., 2021), and, even though it has been little used for preparation of synthetic seeds (Kulus, 2019), seems to have the necessary potential for it.

Inducer of resistance to diseases caused by phytopathogens: chitosan is a recognized elicitor of the acquired resistance system of plants (Singh et al., 2018) through a phenomenon known as defense priming. Studies on these topics include the induction of suberization during the wound healing process in potato tubers (Liu et al., 2019b), the production of secondary metabolites, including phytoalexins (Eilenberg et al., 2010), lignin (Ali et al., 2014), phenolic compounds (Acemi et al., 2021), among others. These activities have been reported to be controlled by MW and DD of the applied chitosan (Hua et al., 2019).

Growth biostimulant: chitosan has been extensively explored in agriculture as a potent bio-stimulant, with many successful applications (Shahrajabian et al., 2021). Thus, its effect on the development of the root system under water deficiency is noticeable (Almeida et al., 2020). Likewise, chitosan remarkably promotes the production of secondary metabolites in plants, i.e., the menthol production in mint crops (Goudarzian et al., 2020). Other successful applications include the foliar treatment of tomato crops under salt stress, using solutions prepared with citric or ascorbic acid, which increases the production of some metabolites and osmolytes, thus helping to reactivate their developments (Attia et al., 2021).

Postharvest protection: chitosan applications can simultaneously fulfill diverse functions such as antimicrobial agent, elicitor, physical isolation (given its ability to form semi-permeable films) (Romanazzi et al., 2018). Likewise, chitosan mixtures with other natural products have successfully been studied seeking synergistic effects and reducing the use of synthetic substances, i.e., chitosan/Aloe vera gels to delay the post-harvest decay of mango fruits (Shah and Hashmi, 2020) and chitosan/carnauba wax/oregano oil to protect cucumber (Gutiérrez-Pacheco et al., 2020).

Finally, to close this brief section on chitinous materials and their main agricultural uses, it is necessary to mention their oligomeric derivatives, better known as "chitooligosaccharides" (COS). These materials have attracted attention in recent years due to the development of new methods to prepare some of them with controlled chemical structures (Sreekumar et al., 2022), which has opened the door, with great expectations, for the understanding of which structures induce specific bioprocesses in pathogens and plants, as well as in other areas such as medicine, i.e., for the treatment of cancer (Sun et al., 2024).

## 3 PGPBs and chitosan to promote plant growth under stress conditions

**Figure 3:**
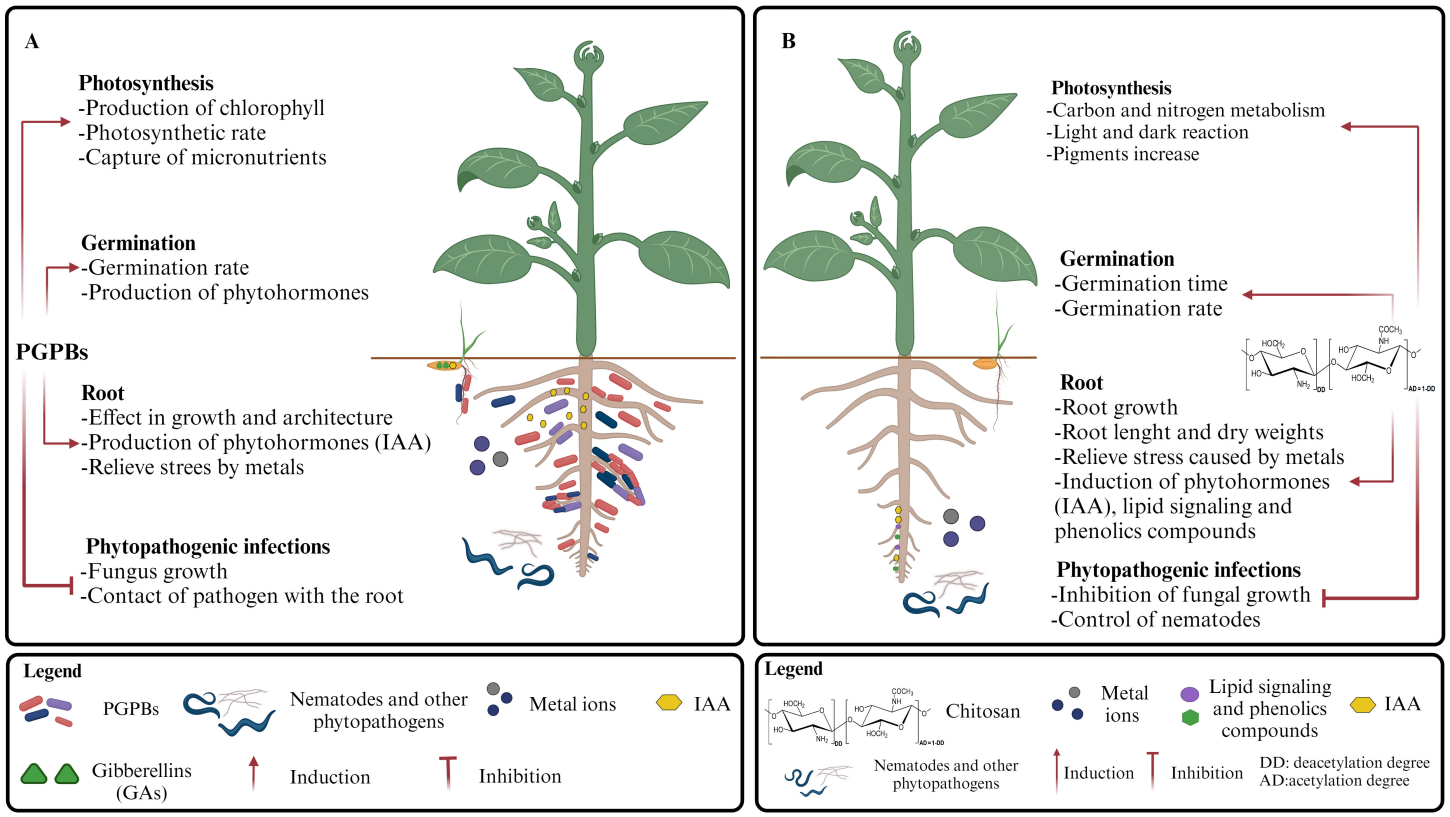
Plant growth promotion processes reinforced by **(A)** PGPBs and **(B)** chitosan.

Some PGPBs generate more specific responses than those mentioned in the previous section, i.e., the production of exo-polysaccharides and/or compatible osmolytes such as sugars (threose), amino acids (proline), betaines, etc*.*, to preserve cellular osmotic balance (Selim et al., 2019). Usually, these PGPBs are known for their capacity to enhance plant tolerance to various types of common abiotic stresses. Likewise, chitosan has shown positive effects on the growth of plants under stress conditions, and its beneficial effects have been reported to alleviate saline stress when used as part of nanomodified biomaterial systems (Balusamy et al., 2022). Similarly, osmo-protection effects have been reported during chitosan foliar treatment of *Puccinellia distans*, a grass that usually grows in high salinity conditions. These positive effects were more noticeable under drastic saline conditions (Oddo et al., 2019). Some comparisons of the effects of chitosan and PGPB on processes associated with promoting plant growth are shown in **Figure 3**.

### 3.1 Photosynthesis

Under stress conditions, the plant photosynthetic process can be severely affected by secondary osmotic and oxidative stress, decreasing transpiration and gas exchanges, and damaging membranes and cell organelles, altering the electron transport process and further generating reactive oxygen species (Hasanuzzaman et al., 2021). Thus, in drought conditions, plants experience a decrease in their chlorophyll content (Ghotbi-Ravandi et al., 2014), among other factors; this triggers an upregulation mechanism to synthesize chlorophyll in greater quantities as a usual strategy of plants to increase their tolerance to drought while reduces the amount of recent photosynthates exported to other organs (Fang and Xiong, 2015). This, in turn, causes changes in the plant organ's metabolic profile, as evidenced in various studies. In this sense, Tripathi et al. (2016) have identified up to 163 metabolites changing their abundance in the roots of plants subjected to water stress after removal from a hydroponic system. From the foregoing, it can be inferred that a diversity of compounds participates in processes such as the maintenance of photosynthesis and cell osmolarity, the slowing down of leaf senescence, and the promotion of root growth, which allow the plants to survive under stress conditions. Moreover, the different allocation of photosynthates, along with the need for osmoregulation, leads to a re-modulation of metabolic pathways. Among the new compounds synthesized and accumulated, sugars, polyols, amino acids, alkaloids, ions, etc*.*, can be mentioned, which are usually produced by upregulated processes (Naylor and Coleman-Derr, 2018). Likewise, an increase in the synthesis of polymers in the cell walls is also generated, which helps to strengthen them and maintain cell turgor (Le Gall et al., 2015). Accordingly, some transcriptomic studies have identified numerous cell wall modifier genes, including some related to xylotransferases involved in the biosynthesis of xyloglucans, which appear upregulated in plants under stress (Tenhaken, 2015). Other biopolymers such as expansin, pectin, lignin, and suberin have also been reported to be upregulated under such conditions (Naylor and Coleman-Derr, 2018).

Some authors have reported the beneficial effect of PGPB on photosynthesis, mainly due to the stimulation of chlorophyll production and photosynthetic efficiency in plants (Erman et al., 2022; Katsenios et al., 2022). In addition, PGPBs produce siderophores that facilitate the capture of metal micronutrients, such as iron (Fe^3+^) and phosphorus (P) (Souza et al., 2015), essentials for the photosynthesis process (Schmidt et al., 2020; Kayoumu et al., 2023) (**Figure 3A**).

Regarding the chitosan effects on photosynthesis, it has been proposed that this biopolymer and its oligosaccharides improve photosynthesis by increasing the activities of various enzymes related to carbon and nitrogen metabolism, as well as the light-dependent and -independent photosynthetic reactions (Ahmed et al., 2020) (**Figure 3B**). Likewise, it has been reported that chitin and chitosan hexamers promote a higher rate of photosynthetic reactions (Li et al., 2020a). In the case of the Fabaceae known as fenugreek (*Trigonella foenumgraecum*), pretreatment of the seeds with chitosan mitigates the effect of salt stress during plant growth, as it was determined by assessment of the relative water content and photosynthetic pigments (Mosapour-Yahyaabadi et al., 2016). Nevertheless, some contradictory results have also been reported regarding the effects of chitosan on photosynthesis, which could be attributed to some factors such as the method and timing of treatment application (Hidangmayum et al., 2019), which are critical in agricultural applications of this material. Likewise, it is also important to consider the characteristics of each applied chitosan because these can exert a notable influence on the results obtained. As previously indicated, appreciable differences in bioactivities may be observed in chitosan of a “similar” composition (Lárez-Velásquez and Rojas-Avelizapa, 2020).

Further promising positive effects of chitosan on photosynthesis in plants under abiotic stresses include the induction of resistance to conditions of water deficit, as evidenced by the increase in photosynthetic rate and harvest yield in maize plants foliar sprayed with N-succinyl chitosan and N,O-dicarboxy-methylated chitosan, or their combination (Rabêlo et al., 2019). Chitosan has also been used as a carrier for the controlled release of biostimulants to induce plant resistance to different stresses. One such application is the formulation of chitosan-selenium nanoparticles and their application to mitigate cadmium stress in Balsam of Moldavia (*Dracocephalum moldavica*) plants. After treatment, an increase in photosynthetic pigments, among other parameters studied, was observed (Azimi et al., 2021). This study showed, additionally, that even when the application of chitosan alone does not produce beneficial effects, a synergistic effect occurs when the two components are used together in the form of nanoparticles. In addition, chitosan alleviates water stress in plants by inducing the production of secondary metabolites, such as γ-aminobutyric acid (GABA) (Geng et al., 2020), a key factor for osmotic adjustment and antioxidant response in plants subjected to abiotic stresses (Carillo, 2018). GABA synthesis, catalyzed by glutamate decarboxylase (GAD), consumes protons, buffering acidosis and regulating cytoplasmic pH under stress conditions. It acts as a ROS scavenger, playing a crucial role in protecting and stabilizing macromolecules, proteins, and membranes. Moreover, upon relief from stress conditions, it exerts an anaplerotic function via the GABA shunt, being converted into Krebs cycle intermediates, thus enhancing the production of NADH and ATP (Ciriello et al., 2024).

### 3.2 Germination

Seed pretreatment with PGPB can promote germination and seedling development in seedbeds (Widawati and Suliasih, 2018; Raja et al., 2019; Rozier et al., 2019). It is known that phytohormones, such as abscisic acid, IAA, GAs, ethylene, cytokinins, brassinosteroids, etc., produced by plants or PGPBs, significantly affect the germination process either positively or negatively. They interact synergistically thus exerting their effects, i.e., some plant genes are necessary for the activity of a phytohormone, while other genes are activated by phytohormones. Since PGPBs play an important role in the production of phytohormones (Bákonyi et al., 2013), they can be used as effective treatments to promote germination and, therefore, crop production (Miransari and Smith, 2014) (**Figure 3A**). Similarly, the positive effects of chitosan on seed germination have been known for decades (Jiang et al., 1994) and have been extensively documented in recent times (Divya et al., 2019; Li et al., 2019; Godínez-Garrido et al., 2022), including reports with positive results for germination under stress conditions (Odat et al., 2021). Despite this, contrasting findings have also emerged in recent studies, depending on the degree of acetylation and the concentration of chitosan used (Gün Polat et al., 2022). Likewise, it has been found that chitosan can favor some parameters related to rate and time of germination (**Figure 3B**) and has no positive effects on others (percentage of germination), as observed, for example, during the germination of maize seeds under controlled low-temperature stress conditions (Guan et al., 2009).

### 3.3 Effects on the roots

Some PGPBs can significantly affect the growth and root architecture of plants under drought stress, improving the plant's ability to uptake water and nutrients, as also observed in plants treated with bio-products such as chitosan (Hidangmayum et al., 2019) (**Figures 3A, B**). For PGPB, the improvement of the root system could be related to the bacterial production of phytohormones, as reported for some species of bacteria producing IAA in green soybean or mung bean crops (Chandra et al., 2018). However, it is also important to consider the possible effect of other naturally occurring factors, i.e. chitin oligosaccharide present in the soil, able to act as bio-stimulants and exert a positive effect on root development (Winkler et al., 2017). Similarly, various studies have reported the positive effects of PGPBs on enhancing plant growth under conditions of metal stress that are usually found in contaminated soils, which usually affect the root system (**Figures 3A, B**). Some of these studies are summarized in **Table 1**.

**Table 1:** Some studies on plants using chitosan and PGPBs for the relief of stress caused by metals.

Metal	Observations	
	Chitosan	PGPBs
Cd	Foliar spraying with chitosan has a high potential in reducing the damage of Cd stress. Chitosan with a molecular weight of 1 kDa exhibited the highest efficacy in mitigating Cd toxicity in wheat seedlings (Liu et al., 2021).	Compared to the Cd-control, witchgrass plants under Cd stress showed increased biomass and IAA production with reduced Cd accumulation when they were inoculated with PGPB (Begum et al., 2019).
Co	Amending soil impacted with wastewater using chitosan and foliar melatonin treatment highly reduced heavy metal distribution in plant parts, including Co, while improved plant photosynthetic efficiency, growth, yield, and grain nutritional quality (Dradrach et al., 2022).	An unclassified haloarchaeal species strain NRS-31 (OL912833), belonging to Haloferax genus*, was isolated, identified for the first time, and applied to mitigate the Co phytotoxic effects on maize plants (Selim et al., 2022).
Mn		The inoculation of *Bacillus cereus* and *Bacillus thuringiensis* increased the absorption of Mn by *Broussonetia papyrifera* and the concentration of Mn in their aerial parts, showing that the two strains could promote its growth (Huang et al., 2020).
Ni	Chitosan can remarkably alleviate the adverse effects of Ni toxicity in *Calendula tripterocarpa* by lowering the bioavailability of Ni in soils (Heidari et al., 2020).	The Ni toxicity-ameliorating and growth-promoting abilities of *Bacillus megaterium* and *Paenibacillus nicotianae* isolates were demonstrated when applied to wheat (*Triticum aestivum*) plants under Ni stress conditions (Pishchik et al., 2021).
V	Application of radiation-degraded chitosan mitigated damages in seedlings of wheat, barley, and rice under vanadium stress (Tham et al., 2001).	Endophytes extracted from *Eriocephalus africanus* roots enabled a high tolerance of *Brassica napus* for vanadium (Mia, 2017).
Zn	Treatments with irradiated chitosan suppressed Zn transport from root to shoot and reduced Zn-induced damage in barley (*Hordeum vulgare*) plants (Nagasawa et al., 2001).	Co-inoculation with *Sinorhizobium meliloti* and *Agrobacterium tumefaciens* enhances metal phytoextraction increasing *Medicago lupulina* growth under Cu and Zn stress (Jian et al., 2019).

*Although this haloarchaea is not a PGPB, it stands as an example of other microorganisms that can be used to promote plant growth by alleviating biotic or abiotic stress.

Although studies on the effect of chitosan on root growth have yielded contradictory results, some showing a decrease in root growth in crops of plants with adventitious roots (Baque et al., 2012), and opposite results in plants inoculated with the nematode *Meloidogyne incognita* (Khalil and Badawy, 2012). In other studies, it has been proposed that the effect of chitosan on root growth is dependent on the mode of application (dose, frequency, formulation, etc.). Indeed, chitosan represents a useful experimental tool to manipulate root development, among other parameters associated with plant growth (Lopez-Moya et al., 2017). Results from other investigations corroborate this proposition. Studies on *Prunus davidiana* seedlings evidenced that root growth was proportional to the concentration of carboxymethyl chitosan added up to a concentration of 2 g/L, whereas root growth decreased at higher values (Xu et al., 2020). Furthermore, in studies with corn seedlings exposed to cadmium stress, chitosan treatment significantly increased the growth rate of the root system and compensated for its functional impairments (Qu et al., 2019). **Table 1** also summarizes several observations from studies on the efficacy of chitosan in relieving metal stress usually found in contaminated agricultural soils.

### 3.4 Protection against diseases

Among the biotic factors that produce the greatest stress in the plants, fungal diseases pose a significant threat due to the significant losses that they can cause in the yield of the crops, in addition to the potentially toxic compounds produced during their infection (Adeyeye, 2016). A recent study with isolates of endophytic bacteria (*Bacillus velezensis* and *Pseudomonas putida*) demonstrated their significant ability to inhibit the growth of *Magnaporthe oryzae* (Do, 2022), a phytopathogenic fungus responsible for the blast infection in rice, an essential crop for human food security. The positive effects of some PGPBs, such as *Bacillus subtilis*, have also been reported on plants facing biotic stress caused by other plant pathogens such as *Fusarium oxysporum* and *Rosellinia necatrix* (Hashem et al., 2019). *Bacillus subtilis* can generate biofilms on plant roots, and chitosan has excellent filmogenic properties, creating barriers against some plant pathogens (de Oliveira and de Oliveira Junior, 2020) (**Figure 3A**). Additionally, some positive results from PGPB studies have been reported regarding the control of weeds associated with crops such as wheat (Abbas et al., 2017; Mustafa et al., 2019).

Similarly, the fungicidal activity of chitosan has been studied for a long time (Allan and Hadwiger, 1979; Romanazzi et al., 2018). In a study comparing the effects of separate treatments with PGPBs and chitosan on the inhibition of the growth of the pathogen responsible for root rot in tomatoes, under similar growth conditions, high molecular weight chitosan was more effective (El-Mohamedy et al., 2013). Unfortunately, combinations of both treatments were not tested in this study. Furthermore, the antiviral and nematocidal activities of chitosan have also been widely recognized (Abd El-Aziz and Khalil, 2020).

### 3.5 Synergistic effects

At this point, it is important to consider that studies based on combined treatments of chitosan and PGPB have been scarce. However, the few studies carried out have found significant synergistic effects between PGPB and chitosan. These studies have shown that the combination of both not only improves disease resistance but also nutrient absorption and plant growth, as well as resistance to different stress conditions. This combined approach seems to take advantage of the individual properties of both agents to offer greater benefits than those achieved when used individually, offering, in this sense, an effective alternative to the chemicals usually used in current agriculture. Some systems that have shown synergistic effects for chitosan and PGPB are discussed below.

#### 3.5.1 Improved disease resistance

A PGPB consortium, formed by *Azotobacter vinelandii* and *Bacillus megaterium* adsorbed on chitosan nanoparticles (PGPB/npChitosan), alleviates the *Fusarium oxysporum* infection damage in *Solanum lycopersicum* (tomato) and *Citrullus lanatus* (watermelon) plants (Pavlicevic et al., 2023), which has been attributed to the PGPB/npChitosan stimulating increased expression of stress-related genes and the activity of antioxidative enzymes in both plants, compared to treatments with PGPB-free or only npChitosan. Additionally, the observed synergy in this system could be also promoted by the following contributions: (a) PGPBs can directly inhibit pathogens through the production of antimicrobial metabolites and antibiotics (Ngalimat et al., 2021), i.e., Azotobacter species usually produce antibiotics with a structure like anisomycin, a well-known fungicidal agent (Sumbul et al., 2020), as well as other metabolites exhibiting antifungal activity (Chetverikov and Loginov, 2009); in addition, *Bacillus megaterium* is recognized as an antibacterial and antifungal antibiotic producing PGPB (Xie et al., 2021); (b) chitosan reduces the severity of the infection caused by *Fusarium oxysporum* by negatively regulating the expression of at least 62 genes in this pathogen, many of which are involved in processes such as plant cell wall degradation, protein, and DNA biosynthesis, as well as some transport of proteins, has also been reported (Elagamey et al., 2022); in addition, it, acting as a bioinoculant carrier, contributes to greater long-term survival of PGPB, enhancing their competition. In fact, it has been documented that chitosan macro beads supported the bacteria survival for over a year, demonstrating its potential as a material for the development of supporting biofertilizers (Perez et al., 2018; Fernández et al., 2022).

Likewise, PGPB-chitosan treatments have also been evaluated in the clubroot disease, caused by the obligate protist *Plasmodiophora brassicae* Woronin (Kang et al., 2024). Although the bacteria *Paenibacillus polymyxa* ZF129 is known to be effective in controlling clubroot disease, it has limited stability in the soil; interestingly, when *P. polymyxa* ZF129 cells were immobilized in chitosan/carrageenan macro-beads (Chit/Carrg, ratio 1/1) a greater survival rate was observed and the control efficiency of ZF129 against clubroot disease improved markedly, with the disease index decreasing from ~94 to ~22, corresponding to a control efficacy around 76. Also, the control index of disease control, which was significantly higher than treatment with ZF129 culture alone (59.76 ± 4.43 %), showed that macro-beads improved the control efficiency of ZF129 culture on clubroot by 16.57 %. Thus, the primary contribution of Chit/Carrg is the protection of the bacterial inoculum from adverse soil conditions and native microflora, facilitating its successful establishment in the rhizosphere and ensuring its effectiveness for a longer period.

Similarly, the encapsulation of *Bacillus licheniformis* in alginate-chitosan nanoparticles supplemented with rice starch had greater antifungal activity against the *Sclerotium rolfsii* infection of *Capsicum annuum* (chili) plants (Panichikkal et al., 2022). These findings demonstrate that the encapsulates of PGPB bacteria with bioactive polymers such as chitosan are phytopathogen biocontrol systems with promising opportunities in current agriculture.

#### 3.5.2 Optimized nutrient uptake

The combined impact of PGPB and chitosan on nutrient uptake by Zea mays L. (corn) has been investigated by Agbodjato et al. (2016). This study unveiled that the *Azospirillum lipoferum-Pseudomonas fluorescens-Pseudomonas putida-chitosan* combination increases the nitrogen and potassium content in the aerial biomass of maize plants, compared to the control and treatment involving only *A. lipoferum* and *P. fluorescens*. Notably, the nitrogen content in the aerial part of the plants increased up to 41.61% compared to the control, after the soil inoculation with the combination of the three bacteria and chitosan. Furthermore, this treatment also induced an increase in potassium content of 6.34% and 27.16% in the aerial and underground parts of the maize plant, respectively. In summary, this combination of PGPB-chitosan has shown promising results in enhancing the mineral nutrition of maize plants, particularly in nitrogen and potassium uptake, which could be related to the ability of PGPB to fix nitrogen, solubilize phosphate, and enhance the availability of other mineral nutrients, thereby making them more accessible and substantially promoting the plant growth and yield (Hasan et al., 2024). On the other hand, chitosan can contribute to soil structure improvement. Particularly, studies using chitosan with lower MW and higher DD demonstrated to reduce porosity, increase mechanical stability, and enhance the wettability of soils; these effects, linked to the creation of electrostatic bonds and/or adhesive bonds between positively charged chitosan particles and negatively charged soil components, results in heightened mechanical stability and water retention, thereby aiding in the retention and availability of nutrients (Adamczuk and Jozefaciuk, 2022). Alternatively, chitosan and these bacteria could facilitate, independent or combined, nutrient uptake by chelating certain minerals, such as Cu and Mn (Verma and Quraishi, 2021), essential for nutrition from plants (White and Brown et al., 2010).

#### 3.5.3 Alleviating stress

In plants, salt stress induces a variety of physiological and metabolic alterations. Some processes, such as germination, photosynthesis, and growth are significantly affected (Hasanuzzaman and Fujita, 2022). The synergy between chitosan and PGPR significantly enhances plant resistance to salt stress. In a study where chitosan-immobilized *Methylobacterium oryzae* CBMB20 was employed as a bioinoculant to improve *Solanum lycopersicum* plant growth, under salt stress, an effective increasing in the plant dry weight was observed. The oxidative stress induced by salinity was also alleviated by up regulating the activities of antioxidant enzymes. Additionally, the accumulation of osmolytes such as proline was observed, along with a reduction in the excess of some Na+ influx into plant cells under these stress conditions, compared to both the control group and treatments involving only *M. oryzae* CBMB20 (in free form) and only chitosan (Chanratana et al., 2019). These effects could be explained by some of the mechanisms proposed for the relief of salt stress in plants by PGPB and chitosan (Kumar et al., 2020; Alenazi et al., 2024). Thus, chitosan can improve the resistance of different crops to diverse abiotic stresses by modulation of signaling pathways, i.e., the application of nano chitosan in certain crops leads to an increase in antioxidative metabolism, resulting in elevated activities of the antioxidant enzymes such catalase, superoxide dismutase, peroxidase and glutathione, reductase (Quitadamo et al., 2021; Alenazi et al., 2024); in addition, chitosan influences the expression of several genes, including mitogen-activated protein kinases, geissoschizine synthase, and octadecanoid-derivative responsive AP2-domain genes, which play relevant roles in salt stress response and alkaloid accumulation (Hassan et al., 2021). Furthermore, inhibition of pathways such as lipid peroxidation has been proposed as a mechanism by which chitosan alleviates salt stress (Peykani and Sepehr, 2018).

On the other hand, a treatment using *Bacillus thuringiensis* (seed treatment) and chitosan (foliar application) on sweet pepper plant grown under different salinity regimes led to improve some growth factors such as chlorophyll content, chlorophyll fluorescence parameter, fruit yield, and to reduce lipid peroxidation and electrolyte leakage in stressed plants; similarly, the proline accumulation and enzyme activity was regulated as well as increased the number of fruit plant, fruit fresh weight plant, and total fruit yield of sweet pepper grown under saline conditions (ALKahtani et al., 2020).

#### 3.5.4 Stimulation of root growth

The combined application of PGPR and chitosan has been evaluated in some studies (Mukta et al., 2017; Ortega-García et al., 2021). For instance, studies involving plants of *Asparagus officinalis L*., bio-fertilized with a combination of the halobacteria-PGPB *Bacillus amyloliquefaciens* and chitosan, showed a remarkable increase in the size and weight of the roby approximately 69% and 25%, respectively, compared to non-treated control and individual treatments of *B. amyloliquefaciens* and chitosan This demonstrates that the synergy between *B. amyloliquefaciens* and chitosan positively influenced vegetative development. Part of these outcomes are attributed to the capability of *B. amyloliquefaciens* to produce growth-promoting substances (Ortega-García et al., 2021), particularly hormones such as gibberellic acid, auxin, and IAA, as well as volatile organic compounds like 2,3-butanediol and acetoin, associated whit the modulation of some processes such as cell division and expansion, as well as promoting lateral root development (Chen et al., 2007; Luo et al., 2022). In turn, chitosan, also induces an augmentation in the length of the primary root, along with increases in fresh weight and dry weight of the root, and, additionally, it has been reported to regulate the root architecture system by increasing the diameter of the root tip and root forks (Jiao et al., 2024). These structural changes imply that chitosan accelerates the development of the root system, thereby improving the absorption rate and transport capacity of water and nutrients necessary to sustain normal plant growth. It is also important to note that chitosan can induce the production of some hormones as IAA and GA in the roots. Moreover, the induced accumulation of IAA may modulate the expression of other genes involved in tryptophan biosynthesis (Mukarram et al., 2023), an amino acid known to promote the development of adventitious roots (Bergonci and Paponov, 2022). Thus, taking everything together, it seems clear that PGPB and chitosan act synergistically to promote the development of a more extensive root system, which is also more efficient in the absorption of nutrients, thus facilitating plant growth.

## 4 Trends in the use of PGPBs and chitosan to increase plant growth and tolerance to diverse types of stress

**Figure 4:**
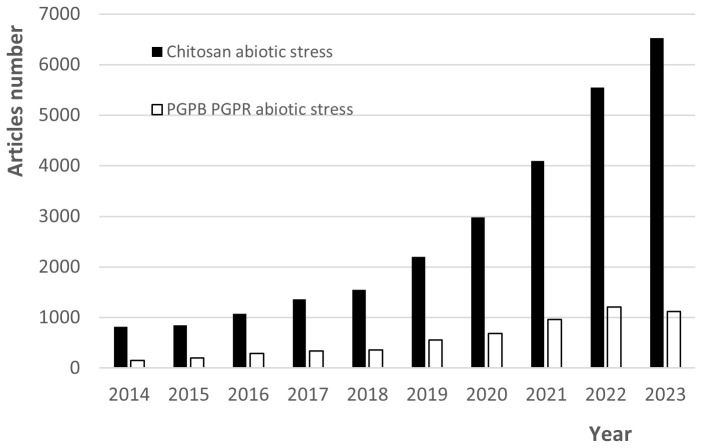
Trends over 10 years for the number of annual papers found in the database “Google Scholar” when “chitosan abiotic stress” and “PGPB PGPR abiotic stress” were used as search words (updated 22 April 2024).

There is currently a strong trend toward the study of biostimulants to promote plant growth, which includes PGPBs and chitosan due to their potential to enhance plant growth under biotic and abiotic stress conditions. **Figure 4** depicts this pattern for the last ten years, showing a significant increase in the number of annual papers retrieved from the Google Scholar database when “chitosan abiotic stress” and “PGPB PGPR abiotic stress” were used as search words. Due to the potential of both to improve the resistance of plants growing under stress conditions, their interactions to improve the response of plants to such conditions researchers have begun to attract attention (Riseh et al., 2021). Furthermore, there is interest in designing green systems for the encapsulation of microbial inoculants using chitosan and other polysaccharide matrices (Perez et al., 2018; Vassilev et al., 2020).

On the other hand, although there are only a few reported studies on the combined applications of PGPB and chitosan as plant growth promoters, their positive results permit to augur a promising near future for these combinations. In this regard, the wide range of possibilities that chitinous biomaterials offer for the development of innovative formulations (in addition to allowing different application methods) as well as the growing expansion of studies related to the impacts on plants of microorganisms obtained from various environments, known, or recently discovered, make this a very exciting field of research.

## 5 Concluding remarks

The above considerations allow infer that the combined treatments of chitosan with PGPBs, and other microorganisms, may enable new strategies to promote plant growth, especially since there is a wide range of possibilities for such combinations, encompassing chitin and chitosan, and including many of its derivatives, as well as different PGPBs. For the application of chitin and chitosan, variations of their traditional parameters could be considered, such as their origin, methods of preparation and chemical modification, molecular weights, degrees of acetylation or substitution, distribution of the N-acetyl groups along chain length, acids used for its dissolution, application methods (powder, solutions, spray, hydrogels, encapsulation matrix for microorganisms, etc.). Its triple activity as an antimicrobial agent, elicitor and film former should also be considered because it guarantees a good initial level of effectiveness.

Regarding PGPBs, the studies related to its effects on plant growth seem to be expanding, especially due to ongoing research to test on plants the impacts of microorganisms derived from different environments, whether known or recently discovered. Importantly, some studies have also shown that PGPB populations can also be significantly reduced in soils under adverse conditions, prompting new strategies to protect them. Among these strategies, the encapsulation of PGPBs using biomaterials enabling them to retain their viability and stability during the production, storage, and application processes in agricultural settings, has emerged as one of the most efficient approaches to protect PGPBs after inoculation into the soil. In this context, the encapsulation of microorganisms useful in agricultural applications, i.e., by using polysaccharides such as chitosan and its derivatives (Rojas-Pirela et al., 2021; Meyer-Déru et al., 2022), emerges as one of the most active areas of research in the foreseeable future, given the importance of the topic and, especially, because its role could be crucial for food production in extreme conditions, a scenario with increasing possibilities shortly according to the projections associated with climate change.
